# Immune Responses to RHAMM in Patients with Acute Myeloid Leukemia after Chemotherapy and Allogeneic Stem Cell Transplantation

**DOI:** 10.1155/2012/146463

**Published:** 2012-06-06

**Authors:** R. Casalegno-Garduño, C. Meier, A. Schmitt, A. Spitschak, I. Hilgendorf, S. Rohde, C. Hirt, M. Freund, B. M. Pützer, M. Schmitt

**Affiliations:** ^1^Department of Internal Medicine III, University of Rostock, 18057 Rostock, Germany; ^2^Cellular Immunotherapy, Department of Internal Medicine V, University Clinic Heidelberg, 69120 Heidelberg, Germany; ^3^Department of Vectorology and Experimental Gene Therapy, Biomedical Research Center (BMFZ), University of Rostock, 18057 Rostock, Germany; ^4^Department of Internal Medicine C, Hematology and Oncology, University of Greifswald, 17487 Greifswald, Germany

## Abstract

Leukemic blasts overexpress immunogenic antigens, so-called leukemia-associated antigens like the receptor for hyaluronan acid-mediated motility (RHAMM). Persistent RHAMM expression and decreasing CD8^+^ T-cell responses to RHAMM in the framework of allogeneic stem cell transplantation or chemotherapy alone might indicate the immune escape of leukemia cells. In the present study, we analyzed the expression of RHAMM in 48 patients suffering from acute myeloid leukemia (AML) and myelodysplastic syndrome (MDS). Furthermore, we correlated transcripts with the clinical course of the disease before and after treatment. Real-time quantitative reverse transcriptase polymerase chain reaction was performed from RNA of peripheral blood mononuclear cells. T cell responses against RHAMM were assessed by tetramer staining (flow cytometry) and enzyme-linked immunospot (ELISPOT) assays. Results were correlated with the clinical outcome of patients. The results of the present study showed that almost 60% of the patients were RHAMM positive; specific T-cells recognizing RHAMM could be detected, but they were nonfunctional in terms of interferon gamma or granzyme B release as demonstrated by ELISPOT assays. Immunotherapies like peptide vaccination or adoptive transfer of RHAMM-specific T cells might improve the immune response and the outcome of AML/MDS patients.

## 1. Introduction

Approximately 80% of patients with acute myeloid leukemia (AML) reach a complete remission (CR) after chemotherapy. However, half of the patients in CR relapse and only 25% of all AML patients survive more than five years. Therefore, there is a fervent need for novel therapies to treat leukemia including immunotherapeutic approaches. Leukemic blasts overexpress proteins that play an important role in survival and proliferation of the cells. These proteins have been designated leukemia-associated antigens (LAAs). LAAs comprise a broad group of proteins including the receptor for hyaluronic-acid-mediated motility (RHAMM) [1]. CD8^+^ T-cell responses against RHAMM have been identified in AML patients [2]. Nevertheless, it remains to be elucidated why immunogenic LAAs are expressed but not sufficiently recognized, and why LAA^+^ malignant cells are not subjected to lysis through specific CD8^+^ T cells. Immune escape might become effective through downregulation of LAAs or hampering of the proper function of T cells [3]. Only limited information is available on the expression of LAAs before and after chemotherapy and/or allogeneic stem cell transplantation (allo-SCT). Therefore we investigated here the expression of RHAMM as well as the frequency of RHAMM-specific CD8^+^ T lymphocytes before and after chemotherapy/allo-SCT. As a future perspective, rather poor immune responses to RHAMM might be enhanced through immunotherapeutic approaches such as peptide vaccination and adoptive transfer of specific T-cell responses in the context of chemotherapy and allo-SCT [1].

## 2. Material and Methods

### 2.1. Blood Samples from Patients and Healthy Donors

We collected 173 peripheral blood samples from 48 patients suffering from AML/MDS after obtaining the patients' informed consent. This study was approved by the local ethical committee. Serial peripheral blood samples were collected at diagnosis, after chemotherapy and/or after allo-SCT with immunosuppression, in CR or during maintenance therapy at sequential time intervals during follow-up and at relapse. Both peripheral blood mononuclear cell (PBMC) and bone marrow mononuclear cell (BMMC) samples were prepared using Ficoll Biocoll separating solution (Biochrom, Berlin, Germany) and cryopreserved according to standard protocols. PBMC samples were obtained from 10 healthy donors and used as negative controls.

### 2.2. Real-Time Quantitative Reverse Transcriptase Polymerase Chain Reaction (RQ-RT-PCR)

 RNA was isolated from a minimum of 2 × 10^6^ cells using RNeasy plus minikit (QIAGEN, Düsseldorf, Germany). Five hundred nanograms of RNA were reversely transcribed into cDNA using the iScript cDNA synthesis kit (BioRad, Munich, Germany). The reverse transcription (RT) products were diluted with 70 *μ*L of molecular-biology water (Sigma-Aldrich). Nine micro liters was used per well. Primers/probes (TaqMan Gene Expression Assays, Invitrogen) were diluted in TaqMan 2xPCR Master Mix according to manufacturer's instructions. Standard curves for RHAMM and ABL were established for each run of RT-PCR using four dilution steps per gene. Copy numbers were calculated by http://www.endmemo.com/. Reactions were tested in duplicate using the ABI PRISM 7900 sequence detection system (Applied Biosystems) and standard conditions with 40 cycles of amplification in 20 *μ*L of volume.

### 2.3. Mixed Lymphocyte Peptide Culture (MLPC)

MLPC was performed as described elsewhere [4]. Briefly, specific CD8^+^ T cells were selected from PBMCs and BMMCs by magnetic-activated cell sorting (MACS) columns (Miltenyi). CD8^−^ fraction was irradiated with 30 Gy and loaded with test or control peptides (20 *μ*g/mL) or cultured with medium alone (no peptide). Peptide sequences of RHAMM and control peptides derived from phosphoprotein-65 of the cytomegalovirus (CMVpp65) and influenza matrix protein (IMP) were ILSLELMKL, NLVPMVATV, and GILGFVFTL, respectively. CD8^+^ and CD8^−^ cells were cocultured in a ratio of 1 : 4. MLPC was supplemented with 10 U/mL IL-2 (Sigma Aldrich) and 20 ng/mL IL-7 (Miltenyi) on day 1. Cytotoxic T lymphocytes (CTLs) were harvested on day seven for enzyme-linked immunospot (ELISPOT) assay and/or flow cytometry analysis when sufficient numbers of CD8^+^ cells were collected.

### 2.4. Mini-MLPC

The MLPC approach was modified into a mini-MLPC in case that insufficient numbers of CD8^+^ cells were obtained from MACS separation. Mini-MLPCs were performed in round-bottom 96-well microtiter plates in RPMI-1640 culture medium supplemented with 10% heat-inactivated human AB serum, 10 U/mL IL-2, and 20 ng/mL IL-7. The ratio was maintained as in the MLPC (1 × 10^4^ CD8^+^ and 4 × 10^4^ CD8^−^ cells, 1 : 4). Number of cells per well was based on the work by Distler et al. [5]. Proliferation observed in mini-MLPCs was comparable to proliferation in conventional MLPCs.

### 2.5. ELISPOT for Interferon Gamma (IFN-*γ*) and Granzyme B

IFN-*γ* and granzyme B ELISPOT assays were performed as described elsewhere [4] to determine specific recognition of RHAMM peptide-positive target cells according to manufacturer's instructions (BD, San Diego, USA).

### 2.6. Flow Cytometry Analysis

The frequency of RHAMM-specific T cells was determined by flow cytometry. Tetramer staining was performed as described previously [2]. Briefly, lymphocytes were stained with RHAMM-specific tetramers and subsequently with conjugated antibodies to CD3 and CD8 (BD Biosciences). Fluorescein isothiocyanate (FITC), peridinin-chlorophyll protein (PerCP), and phycoerythrin (PE) were used as fluorochromes. A minimum of 2 × 10^4^ cells were acquired. Flow cytometry was performed on a Calibur cytometer (BD Biosciences). Appropriated isotype controls were included in each experiment. Data were analyzed using the flow cytometry analysis software FlowJo (Tree Star, Inc, USA). The frequency of tetramer CD8^+^ T cells was considered positive if it was 2-fold or higher than the frequency of CD8^+^ cells counterstained with a tetramer recognizing an irrelevant peptide.

### 2.7. Clinical Status of the Patients

 The clinical status of patients was obtained from the clinical data base of the Department of Internal Medicine III, University of Rostock. Clinical features of the patients such as chimerism analysis, cytogenetics, HLA, CMV status, and therapy were evaluated by the Department of Internal Medicine III, University of Rostock. The FLT3 status was assessed at the Department of Hematology/Oncology at the University of Greifswald, Greifswald, Germany. AML cases were classified according to the FAB criteria and characterized at the cytogenetic level. CR was defined according to standard criteria.

### 2.8. Statistical Analysis

 Statistical analyses were performed using Stat Graphics Plus 5. The standard Wilcoxon signed-rank test was used for nonparametric comparisons of median expression of RHAMM before and after treatment, as the data were paired and not normally distributed. Mann-Whitney *U-*test was used for nonparametric comparisons of median expression of RHAMM in healthy donors and patients. Statistical significance was considered if the *P* value was <0.05.

## 3. Results

### 3.1. Patients' Characteristics

We screened 48 AML/MDS patients in a prospective study. Twenty-one patients received allo-SCT, whereas 27 received only chemotherapy under conventional protocols. Our cohort of patients maintained a ratio of almost 1 : 1 between male (*n* = 23) and female patients (*n* = 25). A normal karyotype was found in 19 patients, and aberrant karyotype in 17 patients, and a complex karyotype in seven patients. The karyotype of five patients was not accessible. There was no significant difference between the age of men and women (*P* = 0.5) at the time of diagnosis. The mean follow-up time of the patients was 272 days (median: 225 days). Nine patients died from leukemia, and three patients from diseases not related to leukemia, that is, encephalitis, pneumonia, and graft versus host disease.

### 3.2. Expression of RHAMM Transcripts before and after Treatment

**Figure 1:**
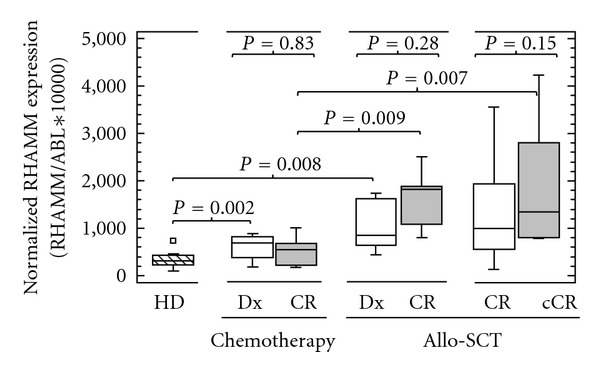
RHAMM expression before and after chemotherapy alone or allo-SCT. Patients at diagnosis had higher copy numbers when compared to healthy donors (chemotherapy group, *P* = 0.002; allo-SCT group, *P* = 0.008). There was no significant difference in expression of *RHAMM *before (white boxes) and after (grey boxes) treatment, neither for chemotherapy alone (*P* = 0.83) nor for allo-SCT (*P* = 0.28), in patients at diagnosis that reached a CR after treatment. Also patients in CR that received allo-SCT showed no difference in *RHAMM *transcripts before (white boxes) and after (grey boxes) transplant (*P* = 0.15). However, patients that received allo-SCT had higher copy numbers when compared to those who received chemotherapy during the CR (*P* = 0.009) or continuous CR (cCR, *P* = 0.007). Medians are shown in the box plots. RHAMM: receptor for hyaluronic acid mediated motility, allo-SCT: allogeneic stem cell transplantation, CR: complete remission, P-value: probability value.

 Expression of *RHAMM* in the peripheral blood of healthy donors (*n* = 10) was very low (median: 318; range 97–730* RHAMM* copies/10^4^ *ABL *copies). In contrast *RHAMM* transcripts were significantly higher in patients before treatment (median: 768; range: 184–36, 160; *P* = 0.001). In order to compare different treatments, patients at diagnosis were split in two groups: patients after chemotherapy alone and patients after allo-SCT (Figure 1). Peripheral blood was collected from 22 AML patients at the time of diagnosis. Thirteen patients were *RHAMM* positive (59%), whereas nine were negative (41%). The expression of* RHAMM* was considered positive when it was higher than the highest value measured in the peripheral blood of healthy donors. There was no significant difference in expression of *RHAMM* in the peripheral blood of patients before treatment according to *FLT3-ITD* status (positive versus negative, *P* = 0.89), gender (*P* = 0.66), or karyotype (normal versus aberrant, *P* = 0.29; normal versus complex, *P* = 0.75; aberrant versus complex, *P* = 0.40).

Furthermore, we aimed to determine the expression of *RHAMM *before and after chemotherapy alone or allo-SCT. Therefore we measured absolute copy numbers of this gene in AML/MDS-diagnosed patients. There was no significant difference before and after treatment, neither by chemotherapy (*P* = 0.83) nor by allo-SCT (*P* = 0.28). However, we observed higher transcript numbers during CR of patients that received allo-SCT when compared to those who received chemotherapy (*P* = 0.009). Furthermore, *RHAMM* was also equally expressed before and after allo-SCT in a cohort of patients in CR. This group also showed higher copy numbers of *RHAMM* after transplantation when compared to the group that was treated with chemotherapy (*P* = 0.007, Figure 1).

### 3.3. RHAMM-Specific CTLs in Healthy Donors

**Figure 2:**
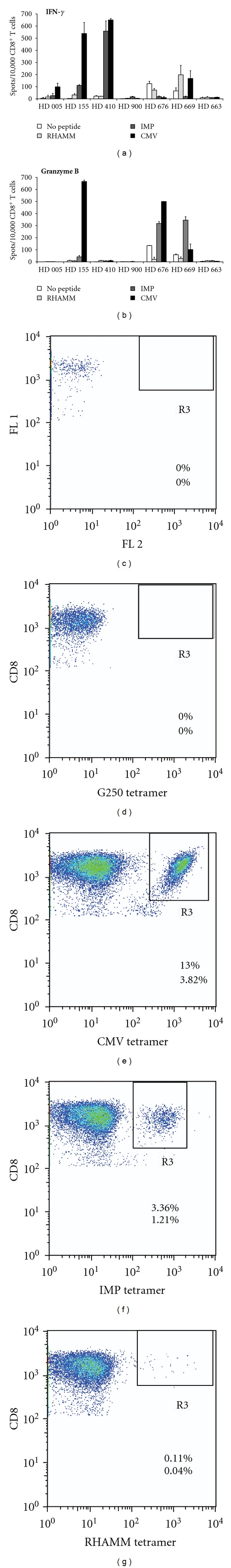
RHAMM-specific CD8^+^ T-cell frequencies in healthy donors. Cells were stimulated in an MLPC for 7 days with different peptides and tested for their reactivity in ELISPOT assays for IFN-*γ* (a) and granzyme B (b) release. Stimulation of cells without any peptide was used as negative control (no peptide), whereas T cells stimulated with CMV and IMP peptides served as positive controls. RHAMM-specific T cells could be detected in two healthy donors (HD 155 and HD 669) by IFN-*γ* ELISPOT, (c)–(g) RHAMM-specific T-cell frequencies were determined by flow cytometry in HD 155. Reported frequencies correspond to gated CD3^+^CD8^+^ T cells (upper numbers), and from all cells in the lymphocyte gate (lower numbers). (c) Fluorescence minus one (FMO) was used as negative control to assess the intrinsic fluorescence of the cells. (d) As a further negative control, cells were cultured in the absence of any peptide and stained with tetramers specific for the irrelevant antigen G250. As positive controls, CD8^+^ T cells were stimulated with either (e) CMVpp65 peptide or (f) IMP-derived peptide, (g) CD8^+^ T cells were stimulated with RHAMM peptide, (e)–(g) CD8^+^ T cells were stained with respective tetramers.

RHAMM-specific T cells were observed in three of ten healthy donors, that is, in HD 155, HD 663, and HD 005, at low frequencies (0.11%, 0.33%, and 0.12%, resp.) with cells in the CD3^+^CD8^+^gate set 100% (Figure 2(g) with controls Figures 2(c)–2(f)). Moreover, an activity of these RHAMM-specific CTLs was detected in two healthy donors (HD 155 and 669) as for IFN-*γ* secretion evaluated by ELISPOT assays (Figure 2(a)). CTLs from healthy donor 669 were not sufficient in number to perform flow cytometry analysis.

### 3.4. RHAMM-Specific CTLs in Patients

**Figure 3:**
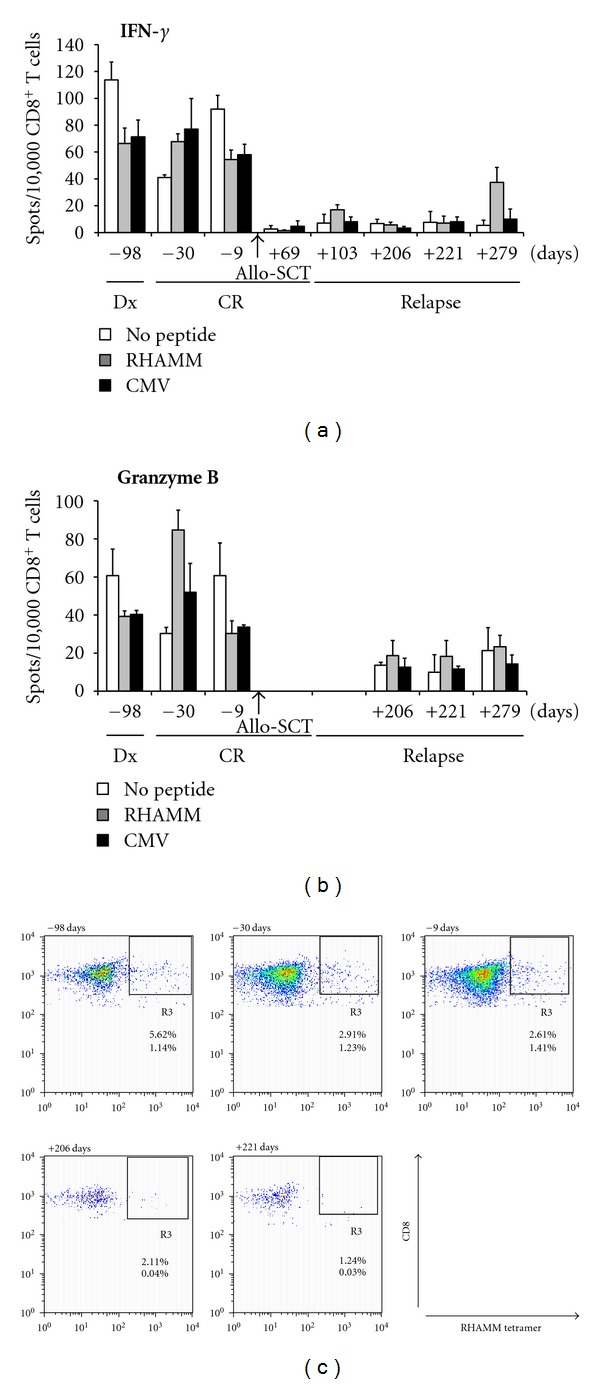
Longitudinal study of RHAMM-specific CTLs in a patient with AML that received allo-SCT. PBMCs were collected from an AML patient at different time points before or after allo-SCT as indicated and subjected to MLPC. CD8^+^ T cells showed RHAMM-specific release of IFN-*γ* (a) and granzyme B (b) at one time point (30 days prior to transplant) when the patient was in CR. This active T-cell population was lost after allo-SCT and not reconstituted when the patient relapsed. (c) CD8^+^ T cells were stimulated with RHAMM peptide and stained with respective tetramer. Frequencies of RHAMM-specific CTLs vanished over the time, as detected by flow cytometry. Reported frequencies correspond to the gate of CD3^+^CD8^+^ T cells (upper numbers) and to all cells in the lymphocyte gate (lower numbers).

Longitudinal studies of RHAMM-specific CD8^+^ T cells were only possible with samples of ten patients who overexpressed RHAMM (29/48 patients) and were HLA-A2 positive, as all peptides used in this study were HLA-A2 restricted. The frequency of RHAMM-specific CD8^+^ T cells in the peripheral blood was measured by flow cytometry during the course of the disease. Furthermore their activation status and potential to kill RHAMM^+^ malignant cells was assessed by secretion of IFN-*γ* and granzyme B, respectively. As displayed in Figure 3(c), RHAMM-specific CTLs were detected at a frequency of 1.24% up to 5.62% of all CD8^+^ T cells, and 0.03% up to 1.14% of all cells in the lymphocyte gate. In ELISPOT assays a general activity of CD8^+^ T cells (Figures 3(a) and 3(b)) was detected at the time of diagnosis (Dx) and in CR. Thirty days prior to allo-SCT a stronger release of granzyme B by RHAMM-R3-stimulated CD8^+^ T cells than by unstimulated T cells was detected. After allo-SCT a general silencing of T cells as for release of both IFN-*γ* and granzyme B was detectable (Figures 3(a) and 3(b)). In accordance with these findings flow cytometry analysis revealed that the number of RHAMM-tetramer^+^ CD8^+^ T cells vanished over the time.

**Figure 4:**
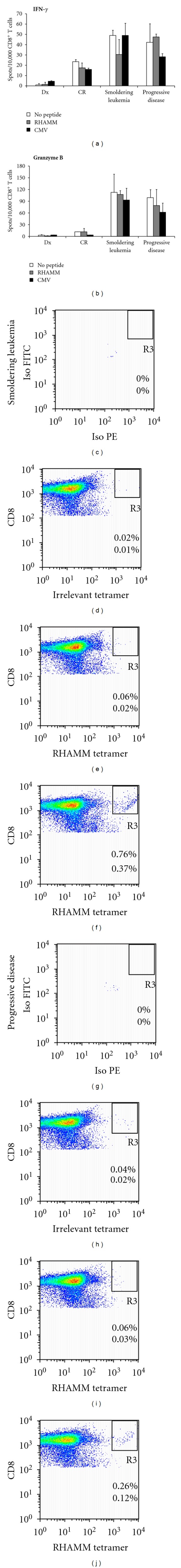
RHAMM-specific CTLs are present, but not functional. PBMCs were obtained from an AML patient who received chemotherapy. (a), (b). RHAMM-specific CTLs detected by flow cytometry at the stage of smoldering leukemia (c)–(f) and at the stage of progressive disease (g)–(j) did not release neither IFN-*γ* (a), nor granzyme B (b) at a level higher than background (no peptide control) as assessed by ELISPOT assays. (c)–(j) Reported frequencies correspond to all cells in the CD3^+^CD8^+^ T-cell gate (upper numbers), and to all cells in the lymphocyte gate (lower numbers). (c), (g) Isotype negative control, (d), (h) Non-peptide negative control, stained with an irrelevant tetramer, (e), (i) Non-peptide negative control, stained with RHAMM tetramer, (f), (j) CD8^+^ T cells were stimulated with RHAMM peptide and stained with RHAMM tetramer.

In another AML patient (Figure 4), RHAMM-specific CD8^+^ T cells were detected by flow cytometry which certainly constituted a distinct subpopulation of RHAMM-specific CD8^+^ T cells (Figures 4(f) and 4(j)), as proven by a number of negative controls (Figures 4(c)–4(e) and 4(g)–4(i)) including RHAMM-tetramer stained CD8^+^ cells which were not stimulated by any peptide (Figures 4(e) and 4(i)). Interestingly we observed a general activation of CD8^+^ T cells at the time of relapse of the patient with no difference of smoldering and progressive disease(Figures 4(a) and 4(b)). This might be due to high concentrations of RHAMM on proliferating malignant cells stimulating specific T-cell responses.

**Figure 5:**
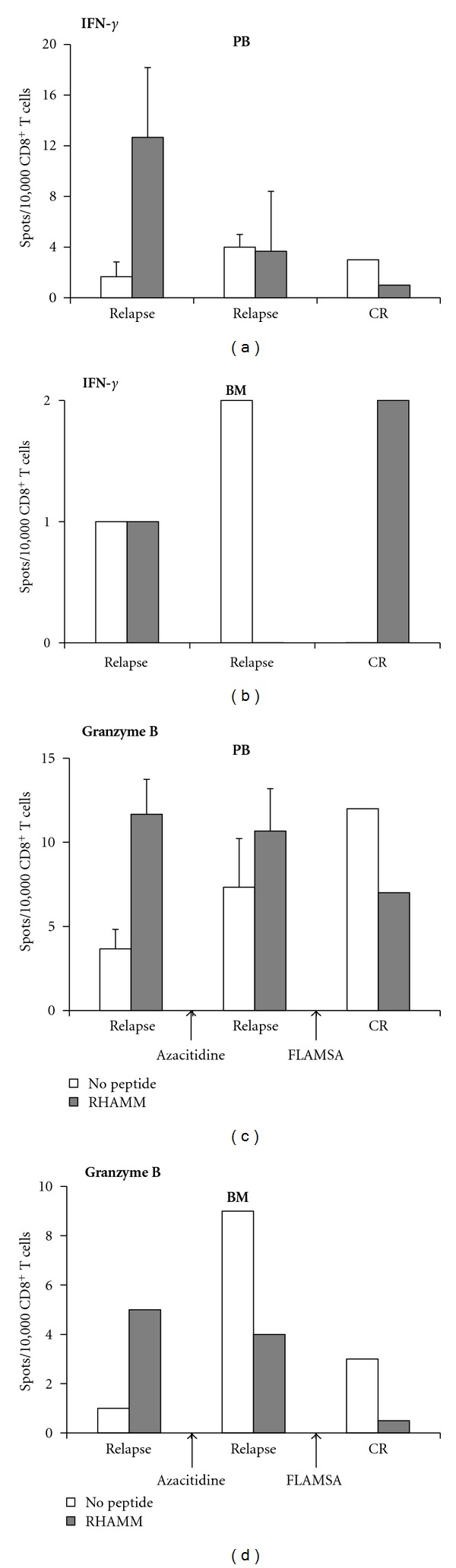
RHAMM-specific CTLs in a patient during relapse of AML. Release of both IFN-*γ* (upper panel) and granzyme B (lower panel) by RHAMM-specific CTLs was detected by ELISPOT in peripheral blood ((a), (b)) and bone marrow ((c), (d)) of a patient at the time of relapse. This population was lost following treatment with azacitidine and conditioning according to the FLAMSA protocol [6] followed by allo-SCT. Cells were not enough to perform flow cytometry analysis. Please note different *y*-axis scaling in this figure.

In a third patient with AML antigen-specific CD8^+^ T cells were detected in both peripheral blood (PB) and bone marrow (BM) to release IFN-*γ* and granzyme B in ELISPOT assays (Figure 5). RHAMM-specific CD8^+^ T cells were able to release IFN-*γ* and granzyme B when the patient relapsed from the disease. This RHAMM-specific CTLs response vanished after therapy with the DNA-methyltransferase inhibitor, azacitidine, and particularly after allo-SCT following a conditioning regimen with fludarabine, amsacrine, and cytarabine (FLAMSA) [6].

## 4. Discussion

In this work we evaluated the expression of RHAMM in 48 patients suffering from AML/MDS. We investigated the expression of RHAMM at RNA level using RQ-RT-PCR in patients before and after treatment. Additionally, we assessed the frequency of specific CTLs for this antigen. These molecular and immunological parameters were correlated with the clinical status of the patients.

RHAMM was firstly described as a soluble binding protein [7]. It is involved in motility, adhesion, proliferation, migration, and angiogenesis [8–11]. RHAMM is also crucial for transformation, metastasis, invasion, growth, and modification of the RAS signaling cascade [12–16]. Multiple forms of RHAMM are overexpressed in a broad variety of solid tumors such as endometrial carcinoma [17], breast cancer [18], pancreatic cancer [19], stomach cancer [20], squamous cell lung carcinoma [21], and malignant melanoma [22], as well as in hematological malignancies like AML, MDS, B-CLL, and multiple myeloma [23, 24]. Seventy percent of AML patients overexpressed RHAMM at both mRNA and protein level [24, 25]. The overexpression of RHAMM mRNA and protein is associated with poor outcome and increased peripheral metastasis in breast cancer patients [26]. Recently, the expression of RHAMM has been reported to be a dismal prognostic factor in AML [27]. RHAMM was identified as one of the most promising LAAs in AML [2, 24]. The nonamer ILSLELMKL (designated R3), position 165–173, is the most immunogenic epitope [4] and it can be naturally processed and presented in an HLA-A2-restricted manner. RHAMM-R3 elicits both humoral and cellular responses in patients with leukemias but not in healthy donors or patients with autoimmune diseases [2, 24, 25]. Moreover, RHAMM-specific CTLs are able to lyse autologous RHAMM*^+^* blasts [4, 28]. Clinical vaccination of AML, MDS, MM, and CLL patients with the RHAMM-R3 peptide elicited specific immunological and hematological responses. Functionally active RHAMM-R3-specific CTLs were detected by tetramer staining in 70% of patients [2, 29, 30]. Peptide vaccination with RHAMM-R3 was safe and effective.

Here we aimed to determine the expression of RHAMM and the spontaneous presence of specific T cells reacting against this LAA in patients with AML/MDS before and after allo-SCT and/or conventional chemotherapy.

Little is known about the prognostic role of RHAMM and its interaction partners in leukemia. Tzankov et al. [27] analyzed RHAMM expression at the protein level on bone marrow biopsies of a large cohort of AML patients. They found that 28% of the patients were RHAMM positive and that RHAMM could be a good prognostic factor at the protein level. However, no systematic study has been done to investigate the role of RHAMM as a prognostic factor at RNA level. To the best of our knowledge this is the first report using RQ-RT-PCR to measure *RHAMM* transcripts and to determine the immune T-cell response before and after allo-SCT. In the present study, we established a robust procedure to quantify absolute copy numbers of *RHAMM *using RQ-RT-PCR. The expression of *RHAMM *was not significantly different with respect to the* FLT3-ITD* status, karyotype, and gender of the patients. In the present study, 59% of the *de novo *AML patients expressed RHAMM. This finding is consistent with work by our group [31]. In our earlier series, 70% (35/50) of the AML patients were positive for *RHAMM* as determined by conventional RT-PCR [31]. Interestingly, some of the patients in the present study who were RHAMM negative at the time of diagnosis tested positive for RHAMM expression during clinical CR. This might be due to reconstitution of hematopoiesis as RHAMM might be also expressed early in stem cell cultures like the other LAA WT1 [32]. Another explanation might be that blasts in the bone marrow of the patient proliferated and therefore overexpressed RHAMM. This may also offer an explanation for the higher copy numbers of *RHAMM *in patients that received allo-SCT and reached CR compared to those treated under conventional chemotherapy.

We hypothesized that the presence of LAAs-specific T cells may be at least in part involved in the maintenance of the CR of patients. Functional RHAMM-specific CTLs were detected by ELISPOT in AML patients (Figures 3 and 5). Nevertheless, this population gradually vanished in the peripheral blood of patients after they received chemotherapy. Potentially downregulating effects of chemotherapy on T cells have been reported previously [6, 33, 34]. Interestingly the frequency of RHAMM-specific T cells increased in the bone marrow which might indicate a trafficking of these cells into the bone marrow [35].

In some of the ELISPOT assays we observed a general activation of CTLs (Figures 3(a), 3(b), 4(a), and 4(b)) which may be at least in part due to an inflammatory cytokine milieu caused by a viral infection.

In summary, *RHAMM* transcripts were indistinctly expressed before and after chemotherapy and allo-SCT. Nevertheless, a clear higher expression of *RHAMM* was observed in those patients at CR that were under allo-SCT. Furthermore, we were able to detect RHAMM-specific CD8^+^ T-cell responses in both healthy donors and AML/MDS patients with overexpression of RHAMM. After chemotherapy and allo-SCT the RHAMM-specific CD8^+^ T-cell subpopulation lost its property to secrete IFN-*γ* or granzyme B and eventually vanished. Therefore the stimulation of this subpopulation by RHAMM-R3 peptide vaccination or the adoptive transfer of RHAMM-specific CD8^+^ T cells might reinstall the antileukemic effect after chemotherapy and allo-SCT.

## References

[1] Casalegno-Garduño R, Schmitt A, Schmitt M (2011). Clinical peptide vaccination trials for leukemia patients. *Expert Review of Vaccines*.

[2] Schmitt M, Schmitt A, Rojewski MT (2008). RHAMM-R3 peptide vaccination in patients with acute myeloid leukemia, myelodysplastic syndrome, and multiple myeloma elicits immunologic and clinical responses. *Blood*.

[3] Rezvani K (2008). PR1 vaccination in myeloid malignancies. *Expert Review of Vaccines*.

[4] Greiner J, Li L, Ringhoffer M (2005). Identification and characterization of epitopes of the receptor for hyaluronic acid-mediated motility (RHAMM/CD168) recognized by CD8^+^ T cells of HLA-A2-positive patients with acute myeloid leukemia. *Blood*.

[5] Distler E, Wölfel C, Köhler S (2008). Acute myeloid leukemia (AML)-reactive cytotoxic T lymphocyte clones rapidly expanded from CD8^+^ CD62L^(high)+^ T cells of healthy donors prevent AML engraftment in NOD/SCID IL2R*γ*
^null^ mice. *Experimental Hematology*.

[6] Chen J, Schmitt A, Chen B (2008). Nilotinib hampers the proliferation and function of CD8^+^ T lymphocytes through inhibition of T cell receptor signalling. *Journal of Cellular and Molecular Medicine*.

[7] Turley EA (1983). Purification of a hyaluronate-binding protein fraction that modifies cell social behavior. *Biochemical and Biophysical Research Communications*.

[8] Till KJ, Zuzel M, Cawley JC (1999). The role of hyaluronan and interleukin 8 in the migration of chronic lymphocytic leukemia cells within lymphoreticular tissues. *Cancer Research*.

[9] Tolg C, Hamilton SR, Nakrieko KA (2006). Rhamm^−/−^ fibroblasts are defective in CD44-mediated ERK1,2 motogenic signaling, leading to defective skin wound repair. *Journal of Cell Biology*.

[10] Slevin M, Krupinski J, Gaffney J (2007). Hyaluronan-mediated angiogenesis in vascular disease: uncovering RHAMM and CD44 receptor signaling pathways. *Matrix Biology*.

[11] Gao F, Yang CX, Mo W, Liu YW, He YQ (2008). Hyaluronan oligosaccharides are potential stimulators to angiogenesis via RHAMM mediated signal pathway in wound healing. *Clinical and Investigative Medicine*.

[12] Hall CL, Yang B, Yang X (1995). Overexpression of the hyaluronan receptor RHAMM is transforming and is also required for H-ras transformation. *Cell*.

[13] Nagy JI, Hacking J, Frankenstein UN, Turley EA (1995). Requirement of the hyaluronan receptor RHAMM in neurite extension and motility as demonstrated in primary neurons and neuronal cell lines. *Journal of Neuroscience*.

[14] Hall CL, Lange LA, Prober DA, Zhang S, Turley EA (1996). pp60(c-src) is required for cell locomotion regulated by the hyaluronan receptor RHAMM. *Oncogene*.

[15] Naor D, Nedvetzki S, Walmsley M (2007). CD44 involvement in autoimmune inflammations: the lesson to be learned from CD44-targeting by antibody or from knockout mice. *Annals of the New York Academy of Sciences*.

[16] Buganim Y, Rotter V (2008). RHAMM in the complex p53 cell cycle network. *Cell Cycle*.

[17] Rein DT, Roehrig K, Schöndorf T (2003). Expression of the hyaluronan receptor RHAMM in endometrial carcinomas suggests a role in tumour progression and metastasis. *Journal of Cancer Research and Clinical Oncology*.

[18] Assmann V, Marshall JF, Fieber C, Hofmann M, Hart IR (1998). The human hyaluronan receptor RHAMM is expressed as an intracellular protein in breast cancer cells. *Journal of Cell Science*.

[19] Aßmann V, Kern HF, Elsässer HP (1996). Differential expression of the hyaluronan receptors CD44 and RHAMM in human pancreatic cancer cells. *Clinical Cancer Research*.

[20] Li H, Guo L, Li JW, Liu N, Qi R, Liu J (2000). Expression of hyaluronan receptors CD44 and RHAMM in stomach cancers: relevance with tumor progression. *International Journal of Oncology*.

[21] Teder P, Bergh J, Heldin P (1995). Functional hyaluronan receptors are expressed on a squamous cell lung carcinoma cell line but not on other lung carcinoma cell lines. *Cancer Research*.

[22] Ahrens T, Assmann V, Fieber C (2001). CD44 is the principal mediator of hyaluronic-acid-induced melanoma cell proliferation. *Journal of Investigative Dermatology*.

[23] Crainie M, Belch AR, Mant MJ, Pilarski LM (1999). Overexpression of the receptor for hyaluronan-mediated motility (RHAMM) characterizes the malignant clone in multiple myeloma: identification of three distinct RHAMM variants. *Blood*.

[24] Greiner J, Ringhoffer M, Taniguchi M (2002). Receptor for hyaluronan acid-mediated motility (RHAMM) is a new immunogenic leukemia-associated antigen in acute and chronic myeloid leukemia. *Experimental Hematology*.

[25] Greiner J, Ringhoffer M, Taniguchi M (2003). Characterization of several leukemia-associated antigens inducing humoral immune responses in acute and chronic myeloid leukemia. *International Journal of Cancer*.

[26] Wang C, Thor AD, Moore DH (1998). The overexpression of RHAMM, a hyaluronan-binding protein that regulates ras signaling, correlates with overexpression of mitogen-activated protein kinase and is a significant parameter in breast cancer progression. *Clinical Cancer Research*.

[27] Tzankov A, Strasser U, Dirnhofer S (2011). In situ RHAMM protein expression in acute myeloid leukemia blasts suggests poor overall survival. *Annals of Hematology*.

[28] Li L, Reinhardt P, Schmitt A (2005). Dendritic cells generated from acute myeloid leukemia (AML) blasts maintain the expression of immunogenic leukemia associated antigens. *Cancer Immunology, Immunotherapy*.

[29] Greiner J, Schmitt A, Giannopoulos K (2010). High-dose RHAMM-R3 peptide vaccination for patients with acute myeloid leukemia, myelodysplastic syndrome and multiple myeloma. *Haematologica*.

[30] Giannopoulos K, Dmoszynska A, Kowal M (2010). Peptide vaccination elicits leukemia-associated antigen-specific cytotoxic CD8^+^ T-cell responses in patients with chronic lymphocytic leukemia. *Leukemia*.

[31] Greiner J, Ringhoffer M, Taniguchi M (2004). mRNA expression of leukemia-associated antigens in patients with acute myeloid leukemia for the development of specific immunotherapies. *International Journal of Cancer*.

[32] Schmid C, Schleuning M, Schwerdtfeger R (2006). Long-term survival in refractory acute myeloid leukemia after sequential treatment with chemotherapy and reduced-intensity conditioning for allogeneic stem cell transplantation. *Blood*.

[33] Seggewiss R, Loré K, Greiner E (2005). Imatinib inhibits T-cell receptor-mediated T-cell proliferation and activation in a dose-dependent manner. *Blood*.

[34] Chen J, Schmitt A, Giannopoulos K (2007). Imatinib impairs the proliferation and function of CD4^+^ CD25^+^ regulatory T cells in a dose-dependent manner. *International Journal of Oncology*.

[35] Mazo IB, Honczarenko M, Leung H (2005). Bone marrow is a major reservoir and site of recruitment for central memory CD8^+^ T cells. *Immunity*.

